# Design and Usability of an Avatar-Based Learning Program to Support Diabetes Education: Quality Improvement Study in Colombia

**DOI:** 10.1177/19322968221136141

**Published:** 2022-11-14

**Authors:** Emma Bishop, Daisy Allington, Tim Ringrose, Clare Martin, Arantza Aldea, Maira García-Jaramillo, Fabian León-Vargas, Yenny Leal, Diana Henao, Ana Maria Gómez

**Affiliations:** 1 Cognitant Group, Oxford, UK; 2 Faculty of Technology, Design and Environment, Oxford Brookes University, Oxford, UK; 3 Faculty of Engineering, Universidad EAN, Bogotá, Colombia; 4 Faculty of Mechanical, Electronic and Biomedical Engineering, Universidad Antonio Nariño, Bogotá, Colombia; 5 Institut d’Investigació Biomèdica de Girona Dr. Josep Trueta, Girona, Spain; 6 Endocrinology Unit, Hospital Universitario San Ignacio, Pontificia Universidad Javeriana, Bogotá, Colombia

**Keywords:** Colombia, diabetes management, education, virtual reality, avatar, human-centered design

## Abstract

**Background:**

This quality improvement study, entitled Avatar-Based LEarning for Diabetes Optimal Control (ABLEDOC), explored the feasibility of delivering an educational program to people with diabetes in Colombia. The aim was to discover how this approach could be used to improve awareness and understanding of the condition, the effects of treatment, and strategies for effective management of blood-glucose control.

**Methods:**

Individuals with diabetes were recruited by Colombian endocrinologists to a human-centered study to codesign the educational program, using the Double Diamond model. Participants contributed to two phases. The first phase focused on gathering unmet educational needs and choice of curriculum. Three prototypes were developed as a result. During phase 2, a different group of participants engaged with the program for several weeks, before reporting back.

**Results:**

Thirty-six participants completed a Web survey during phase 1, and five were also interviewed by telephone. The majority (33 of 36; 91%) were receptive to the prospect of educational interventions and ranked the chosen topic of hypoglycemia highly. In phase 2, the three prototypes were tested by 17 participants, 10 of whom also gave feedback in focus groups. The response was overwhelmingly positive, with 16 of 17 (94%) stating they would use a program like this again. The 3D version was the most highly rated.

**Conclusions:**

Immersive, avatar-based programs, delivered through smartphone, have the potential to deliver educational information that is trusted, engaging, and useful. Future work includes expansion of the curriculum, evaluation with a larger group, and exploration of the prospective role of artificial intelligence in personalizing this form of educational intervention.

## Introduction

Colombia has the second highest incidence of diabetes of any country in South and Central America, according to the International Diabetes Federation.^1^ Estimates suggest that there were 3.4 million adults known to have the condition in 2021, with a further 1.2 million living with it undiagnosed. Prevalence of the condition is increasing, due to sedentary lifestyles, societal dietary patterns, low educational levels, aging of the population, and the high rate of urbanization.^2^ The risk of developing long-term complications associated with diabetes can be reduced by optimizing glycemia,^3^ but this requires knowledge of a variety of factors, including blood glucose dynamics, medication, and technology. Hence, there is a potential appetite for engaging educational interventions that deliver the skills needed to improve control safely, as evidenced by recent research.^4^ Education has also been linked to multiple key drivers of quality improvement (QI) for people with diabetes (PWD),^5,6^ and there is evidence that some QI strategies can improve outcomes for socially disadvantaged groups.^7^ Hispanic populations are among those who are more likely to have higher levels of acute complications, less optimal glycemia, and less use of technology.^8^ This could result from language or cultural differences, lack of financial resources, or distance from care providers.^9^ Solutions must therefore include equitable access to health care and education.^8^

Emerging technologies, such as mobile games and virtual reality (VR), are proving to be popular and effective educational tools that can overcome language, literacy, and numeracy barriers, and stimulate new behaviors.^10^ The interactive, visual content can improve recall and retention of information.^11^ Avatar-based technology can have a positive effect on knowledge and self-care among people with chronic conditions, such as diabetes.^12^ Such technology can be used to create easy-to-understand, interactive health care information in 3D, which individuals can view on smartphones, iPads, or VR headsets, to learn how to self-manage their health. The content is prescribed by clinicians and can be viewed both in the clinic and at home. This approach has potential to provide timely, trusted information in a country like Colombia, where 85% of the population live in areas covered by 3G/4G and 63% own smartphones,^13^ at a time when mobile phones are transforming the landscape of diabetes care around the world.^14^

Cognitant Group Ltd (Oxford, UK) was the lead partner in the Avatar-Based LEarning for Diabetes Optimal Control (ABLEDOC) project, which brought together a multidisciplinary team of academics, clinicians, and industry professionals from the United Kingdom and Colombia to explore the feasibility of an avatar-based educational program for PWD in Colombia. Virtual reality education is safe and well-liked among clinical diabetes staff,^15^ but this is the first study of its use with PWD in Colombia, according to a PubMed search. The aim of this QI study was to work collaboratively with a small group of Colombian clinicians and PWD, using a structured method to fully understand the local problems and current educational practices, to discover how such an educational intervention might improve awareness and understanding of the condition, the effects of treatment, and strategies for effective self-management. A pilot program for PWD in Colombia was subsequently developed, delivered, and evaluated, focusing on an identified intervention from the design study.

## Methods

**Figure 1.**
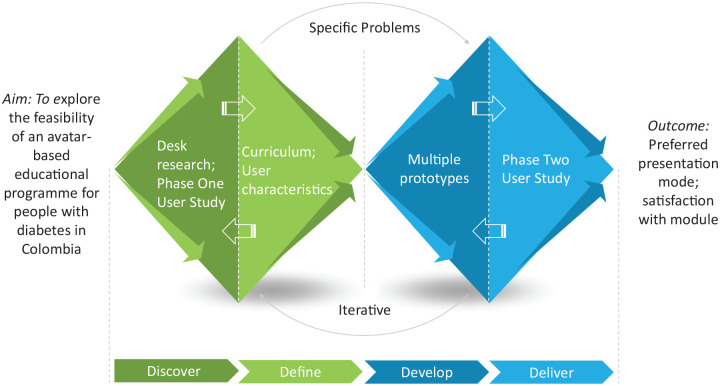
ABLEDOC double diamond design process.^a^ Abbreviation: ABLEDOC, Avatar-Based LEarning for Diabetes Optimal Control. ^a^This figure was designed using a template from PoweredTemplate.com.

The human-centered Double Diamond methodology^16^ was used to understand the local problems and current educational practices for diabetes in Colombia. This process comprises four steps: *Discover, Define, Develop*, and *Deliver*. The steps are separated into two diamonds, each of which has a divergent phase, to expand the problem space, then a convergent phase that narrows down the options (see Figure 1). The first diamond allows researchers to *discover* the problem from the perspective of those most affected by it, instead of relying on assumptions. The resulting insights are used to *define* the challenge. The second diamond encourages co-creation by *developing* different solutions to the clearly defined problem. *Delivery* involves testing the solutions, with a range of people to improve the final result. The steps were instantiated within the ABLEDOC project as follows:

1. *Discover* the educational experience and needs of different demographic groups, as well as key trends that could inform the curriculum, by working with expert clinicians and patient associations in Colombia.
2. *Define* the pilot topic of choice, target audience, and full curriculum.
3. *Develop* multiple prototypes of an avatar-based program for PWD in Colombia, focusing on one educational intervention.
4. *Deliver* the prototypes to a small cohort of participants to evaluate the approach and determine the preferred visual delivery method.

The methodology for each of these phases is defined below. The version described here includes modifications due to the COVID-19 lockdown in Colombia, which occurred during the study period. All of the user interactions that were originally designed to be face-to-face were moved online as a consequence.

### Discover

The discovery phase was divided into two parts: desk research and a user study.

#### Desk Research

The purpose of the desk research was to assimilate information on three aspects of the experience of PWD in Colombia. These were as follows: unmet educational needs, pilot topic of choice, and health information access. The team collected information about which materials are typically received and at what point in the patient journey to learn where the intervention could fit into a traditional educational program and how to disseminate it effectively.

The study team included two endocrinologists from the Hospital Universitario San Ignacio (HUSI; Bogotá, Colombia) and was supported by an external advisory board of clinicians and academics. Key learning needs and a pilot topic of choice were identified from the HUSI clinicians’ observations and assumptions. These were reviewed by the advisory board and used to develop a draft educational curriculum for validation in the phase 1 User Study.

#### Phase 1 User Study

The purpose of this phase was to identify any issues associated with the choice of curriculum, its presentation, and the target group. The study encompassed a Web survey and interviews to obtain a deeper understanding of health information access and topics of interest from the participants’ perspectives.

The phase 1 recruitment target was 35 participants from the HUSI, all of whom were required to provide verbal and written informed consent. Thirty of the participants were invited to complete a 30-minute online survey (group 1), and five were asked to participate in a 1:1, 30-minute telephone interview, conducted by a clinician (group 2). Inclusion criteria were as follows: adult participants aged 18 to 65 years, type 1 diabetes (T1D) or type 2 diabetes (T2D), and a disease duration >1 year. Additional inclusion criteria for group 2 include treatment with a prandial glucose regulator, insulin, and/or a sulfonylurea. All participants were required to be regular smartphone users, with access to an iPhone or an Android phone.

Interview data were collected through audio recording and note-taking. The recordings were subsequently transcribed into Spanish and then translated to English for analysis. The protocols for this and the phase 2 user study were approved by the research and institutional ethics committee of the HUSI.

### Define

The results of the Discover phase were used to define the pilot topic of choice, target audience, and final syllabus.

The survey data allowed investigators to review trends in unmet learning needs and factors that most affect the participants’ quality of life, according to individual characteristics, to refine the curriculum requirements in relation to the pilot topic.

The interview data provided a more complete picture of in-depth personal experiences and patient journeys. This was used to construct personas to give a tangible picture of the lives of the target audience. For example, what they think; how they behave; their wants and needs, along with their fears or frustrations; and their influencers and environment.

The syllabus was characterized in terms of learning objectives and educational approach.

### Develop

The next step was software development. The approved curriculum was transformed into an evidence-based storyboard to deliver engaging content, designed to promote health behavior changes that the participants would be likely to adopt. The software was then created using 3D models, visual animations, and text prompts to aid understanding and recall.

**Figure 2.**
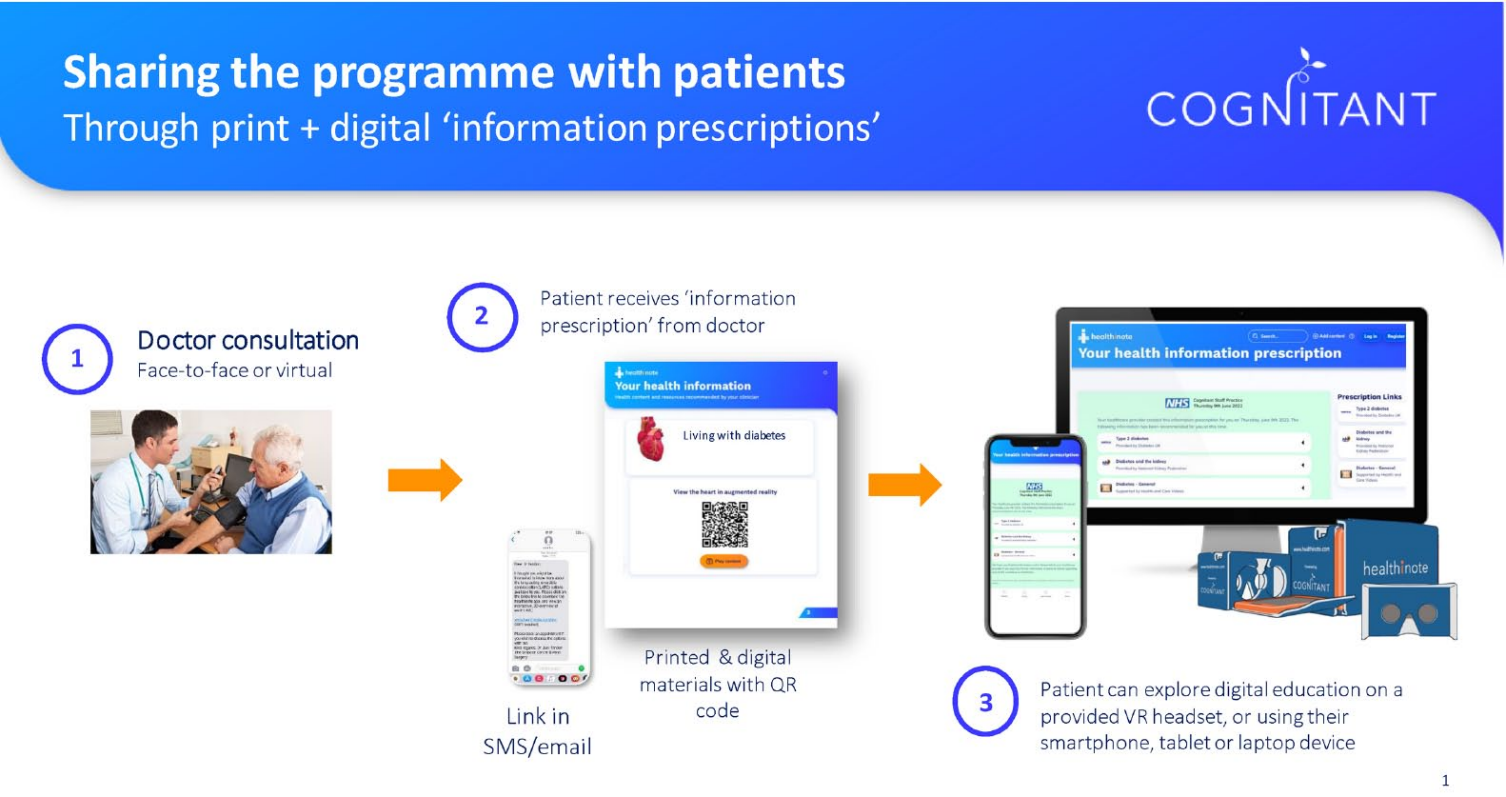
Cognitant’s educational prescription model, via Healthinote. Abbreviation: VR, virtual reality.

Three learning modules were created to allow users to choose which mode of visual presentation was the most effective for their needs. The content was delivered via the *Healthinote* app (Cognitant, Oxford, UK). The workflow through which information is disseminated using Healthinote is shown in Figure 2. Clinicians prescribe educational content, based on individual needs, through a quick response (QR) code or link in an SMS message. Users can then engage with the tailored information by viewing the VR content on their smartphones.

### Deliver

The purpose of the phase 2 User Study was to assess the preferred mode of presentation, satisfaction with modules, increase in knowledge, and confidence to self-manage. The recruitment target was 12 to 15 participants, and inclusion criteria were the same as phase 1, group 2. Participants were given the opportunity to engage with the program over a period of several weeks, before reporting back through a series of three-hour online focus groups. All participants were asked to complete a post-study questionnaire.

At the start of the study, participants received an SMS text with written instructions explaining how to access the program. They were also supplied with a Google Cardboard headset^17^ to view 3D content. The HUSI team created videos describing how to download the Healthinote app and how to use the headset, and Cognitant translated its instruction video to Spanish.

After a minimum of four weeks, participants were invited to join a focus group. Opinions were sought on the choice of topic, method of presentation, use of avatar, and comparison with current access to educational material. Users were also asked to identify any barriers or concerns about the use of this technology.

Data collection was similar to phase 1, with the additional option of supplementing with photos and sketches. Notes and transcripts from both the user studies were translated and analyzed thematically.^18^

## Results

### Discover

#### Desk Research

The HUSI clinicians noted that patients often have health literacy and knowledge limitations, and that 10- or 20-minute consultations (primary and secondary care, respectively) do not provide sufficient time to communicate critical information. They unanimously agreed, together with the advisory board, that hypoglycemia was a key subject area to focus on. Key learning needs were derived from the clinicians’ observations and assumptions (see extract in Table 1).

**Table 1.** Extract From Clinicians’ Observations in Routine Practice in Colombia.

Observation	Assumption	Action
Pilot topic of choice: Hypoglycemia		
• People receiving insulin or certain oral medications often report that they struggle with hypoglycemia (impacting quality of life and HCP time/costs) • Hypo anxiety and weight gain in people receiving such agents • Lack of knowledge and clear need for further support	• People do not always fully understand their condition or medication • People would benefit from an effective educational intervention • Unmet learning needs are: preventing hypos, detecting hypos, treating hypos, dosing, self-care, sick-day rules, impact on driving • People experience hypoglycemia differently, which could make detection more difficult • Educational needs may be different, for example, newly diagnosed versus long-term diagnosis • Hypo anxiety is a significant issue and could be addressed by improving knowledge of their condition and medication	• Pilot module content, language, and flow has been shaped around these challenges • Decided approach/language needs to be calming, hopeful, optimistic, informative • Module to allow for user-led navigation so that people can access information most relevant to them

Abbreviation: HCP, health care provider.

**Figure 3.**
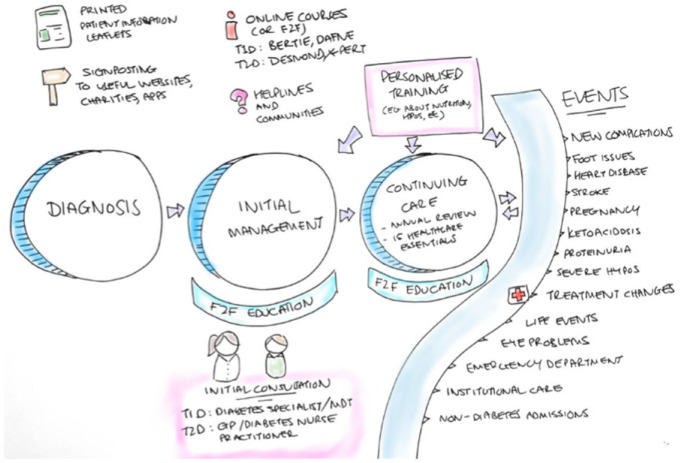
Desk research into health information access in Colombia. Abbreviations: F2F, face-to-face; T1D, type 1 diabetes; T2D: type 2 diabetes.

People in Colombia receive information through a variety of sources, including face-to-face education, printed leaflets provided by their local hospital, online content, communities, and personalized training, and this continues throughout their journey—from diagnosis through to continued care (as summarized in Figure 3).

#### Phase 1 User Study

A total of 36 participants responded to the Web survey, with a mean age of 54 years (19-82 years). Most people rated their health literacy as high: average understanding was 4.5 (out of 5), regarding both their condition and medication (although the range was 2-5). Eighty-six percent of the cohort had a time since diagnosis of >5 years (see Table 2).

**Table 2.** Participant Demographics: Diabetes Duration, Sex, Type, Smoker, and Treatment.

	Phase 1		Phase 2	
Demographic	n	%	n	%
Time since diagnosis, y				
>5	31	86	15	94
2-5	2	6	1	6
<2	3	8	0	0
Sex				
Male	16	44	5	31
Female	20	56	11	69
Diabetes type				
Type 1	22	61	14	88
Type 2	14	39	2	12
Smoking status				
Occasional smoker	4	11	0	0
Nonsmoker	31	86	16	100
No response	1	3	0	0
Treatment				
Insulin only	25	69	14	88
Insulin and tablets	8	22	2	12
Tablets only	3	8	0	0

Note that percentages are rounded to integers, and one phase 2 respondent declined to provide demographic information.

The majority were well-educated (78% higher education), and 33 of 36 (91%) were receptive to the prospect of educational interventions to help them self-manage (with 58% responding “yes” and 33% “maybe”) when asked, “Do you think you need more information in order to control your condition better?”). Respondents were allowed to select multiple options when asked how they prefer to access health information. Twenty-six participants (72%) selected “Speaking directly with your doctor or nurse,” with 18 (50%) citing “videos, TV shows or computer animations.” When asked about health information sources *other* than doctors, 64% relied on family alone, with a few also citing friends. Eight of 36 (22%) listed support groups, such as social media, and 28% did not rely on anyone apart from themselves. All respondents felt that lifestyle changes can have a positive impact on health, meaning that participant “buy-in” was not necessarily an obstacle for this study to overcome.

Respondents were asked to rank topics of interest from a preset list. Results are shown in Table 3. Hypoglycemia ranked highly, and additional suggestions included nutrition and alcohol consumption. The responses ranging from “very useful” to “not very useful” are grouped by participant characteristic in Table 4. The two respondents who answered “not very useful” were not insulin-dependent.

**Table 3.** Usefulness of Topics as Graded by Web Survey Respondents.

Most useful Least useful	Topic	Score^a^
	How diabetes can affect vision	111
	Self-care (eg, diet, exercise)	111
	Emotional support (eg, depression, anxiety)	110
	Hypoglycemia	109
	How to use medicines	109
	Heart and kidney issues associated with diabetes	107
	Foot and nervous system issues associated with diabetes	107
	Diabetes and infections	107
	Technology	106
	Diabetes and COVID-19	106
	What is diabetes?	104
	Check-ups required if you have diabetes	102
	How diabetes may affect driving	92

a Based on scores: not useful (0), not very useful (1), OK (2), useful (3), and very useful (4) assigned per respondent; therefore, lowest score possible = 0, highest score possible = 144.

**Table 4.** Response, By Characteristic, to the Question “How Useful Do You Consider the Following Topic: How to Treat and Avoid Hypoglycemia?” (n = 36).

	Very useful		Useful		OK		Not very useful	
Demographic	n	%	n	%	n	%	n	%
Sex								
Male	3	25	10	67	3	43	0	0
Female	9	75	5	33	4	57	2	100
Diabetes type								
Type 1	12	100	7	47	3	43	0	0
Type 2	0	0	8	53	4	57	2	100
Treatment								
Insulin	12	100	9	60	4	57	0	0
Insulin and tablets	0	0	6	40	2	29	0	0
Tablets only	0	0	0	0	1	14	2	100
Time since diagnosis, y								
>5	12	100	14	93	4	57	1	50
2-5	0	0	1	7	1	14	0	0
<2	0	0		0	2	29	1	50
Educational level								
Higher education	11	92	12	80	5	71	0	0
High school diploma	1	8	1	7	1	14	1	50
Primary education	0	0	2	13	0	0	1	50
No education	0	0	0	0	1	14	0	0

In telephone interviews (n = 5), participants reported that they were unaware of some problems related to inadequate glucose control, they had encountered issues in their professional and social lives, and were frequently supported by family. The interviews also uncovered unexpected aspects, such as the potential impact of personal time limitations on self-management. Almost all people interviewed thought that a program on hypoglycemia would be very useful, particularly how to manage severe episodes.

### Define

**Figure 4.**
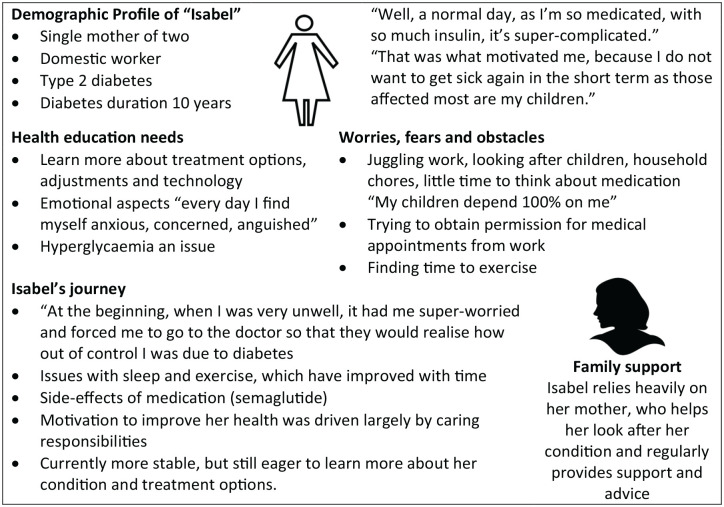
Participant persona, as noted from 1:1 conversations with real-life people with diabetes in Colombia. The name is fictional, but details are based on a real person’s details and quotes (translated from Spanish).

The pilot topic was defined to be hypoglycemia. The target audience was characterized using personas to capture individual demographics, journeys, and health education needs, together with fears and frustrations. An example is shown in Figure 4.

The final output of phase 1 was the definition of the curriculum for the pilot module. The learning objectives and contextual information are shown in Table 5.

**Table 5.** Pilot Module Curriculum.

Learning objectives	
• Improved ability to recognize hypoglycemia • Understanding of how to treat mild-to-moderate hypoglycemia, what to do in severe cases, and how to be prepared for an event • Increased awareness of factors/situations that increase the risk of hypoglycemia and how to avoid these	
Audience	
Age	Adults
Literacy level	Accommodate for low literacy levels by using visual and aural aids, without alienating/patronizing those with higher literacy skills
Issues	• On average, people with type 1 diabetes (T1D) experience up to two episodes of mild hypoglycemia a week and up to two serious events per year^19^ • Severe hypoglycemia is associated with an increased risk of mortality^20^, and associated anxiety may have a significant impact on quality of life^21^ • People may not be aware of what hypoglycemia is, how to recognize symptoms, and/or how to treat an event • At-risk and anxious people may overeat to raise their blood sugar levels, causing weight gain, and they may become reluctant to take their medication as prescribed^22^; both factors increase their risk of diabetes-related complications • Increasing awareness and ability to self-manage may reduce incidence of events and relieve anxiety
Context	People with existing or recently diagnosed T1D or type 2 diabetes (T2D), at risk of/struggling with hypoglycemia, receiving such agents as prandial glucose regulators, insulin, and/or sulfonylureas
Approach	
Language	Nontechnical yet not patronizing
Voice	• Reassuring and positive; 25% of people with diabetes suffer “hypo anxiety”^21^ • Realistic and supportive
Visuals	Educational, interactive, engaging
VR presence	Calming, welcoming, interesting Stylized as opposed to graphic, realistic, or clinical
Music	Calming, clinical/slightly abstract, familiar

### Develop

**Figure 5.**
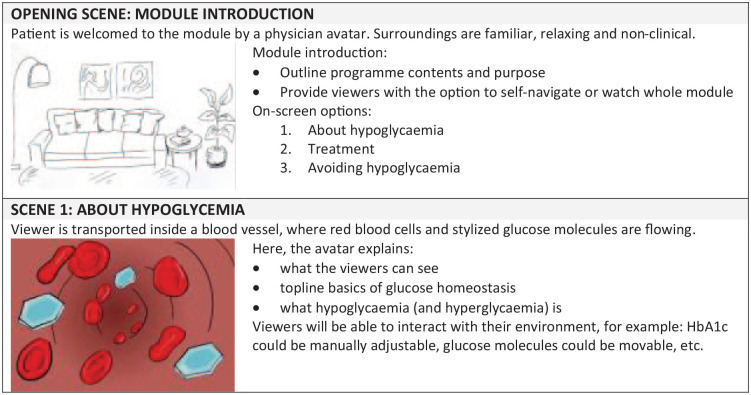
Extract from pilot module story flow. Abbreviation: HbA1c, hemoglobin A1c

The approved curriculum was converted into a story flow to guide the viewer through the content (see Figure 5). A site map was also created to allow viewers to skip or navigate to different scenes at any point during the program. The immersive content was then produced, together with a Spanish script, and narrated by a native Colombian speaker to aid familiarity.

**Figure 6.**
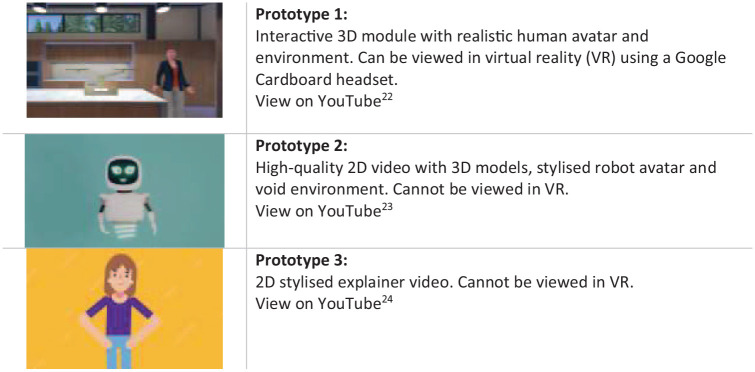
Pilot module prototypes.

In response to results obtained from earlier phases, three pilot programs were developed. Each prototype comprised a three-minute excerpt of a full hypoglycemia program, focusing on causes and symptoms, to rapidly identify a mild to moderate event. Prototypes included exactly the same narration and content, but visual approaches were very different (see Figure 6).

### Deliver

The three prototypes were tested by 17 participants (see Table 2), who gave feedback through the Web survey. All respondents were insulin-dependent, and 88% had T1D. Ten participants also attended one of two online focus groups, each for five people. The data were transcribed and translated into English for analysis.

**Figure 7.**
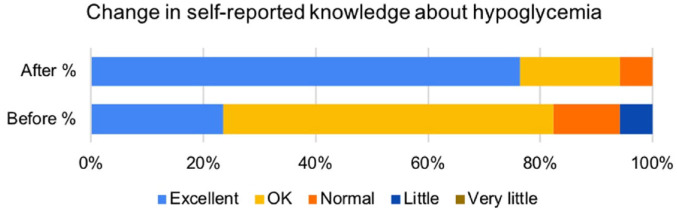
Response to the question, “How would you rate your knowledge about hypoglycemia and how to handle it?” before and after the intervention (n = 17).

In all, 16 of 17 (94%) responded “yes” and one (6%) “not sure” when asked “Would you use a program like this again to learn more about your health?” In response to the question “Did you like the program?” 15 of 17 (88%) said “Yes” and two said “OK.” There was a marked improvement in self-reported knowledge (see Figure 7), and qualitative analysis of comments also revealed a clear pattern of positivity for all three prototypes (see Table 6). All three prototypes were considered to be very interesting, relevant, novel, and comprehensive educational tools. Notably, one person had never been informed as to what hypoglycemia was and had actually been experiencing events and mistaking them for perimenopausal symptoms.

**Table 6.** Indicative Comments From Two Three-Hour Focus Groups, With Regard to the Overall Experience of the Pilot Study.

Topic	Comment
Ease of use	“I have 44 years of being diabetic and I have used a lot of literature. As a child I did not understand and I was bored but now with this technology a child can learn. The videos are very compact and easy to understand, they teach you clearly. Videos like these can be used to educate everyone.”
Visual presentation	“I have had diabetes since I was 5 years old and I have seen everything from books, guides, brochures, group workshops, medical visits. All that is boring and you don’t pay attention, the videos are very friendly and help to gain interest, they involve you with your sight and hearing and educate you better.”
Unmet needs	“I am type 2 diabetic. I found the video excellent and I have learned a lot, I did not know that hypos existed. No one had told me anything. I had all the symptoms described in the video but I did not know what it was and my family told me it was pre-menopause . . . I think it’s great to have a video that can be seen at any time because I don’t have time to read brochures and books.”

Participants provided positive feedback on the vocabulary, visuals, sound, content, and topic of choice. They also reacted favorably toward this mode of delivery and believed that VR holds value as an effective and appealing medium. There were very few technical issues and participants suggested technical enhancements, such as adding augmented reality or animating the avatar to describe symptoms.

The 3D video with a realistic avatar (prototype 1) generated the most positive response in the qualitative data analysis (see Table 7). Participants cited the familiarity of the home environment and the immersive VR element, for example. They were less keen on the robot (prototype 2), commenting that it was “impersonal.” Responses were mixed, however, and participants liked all options, with some describing prototype 3 as “easier to understand.” Additional topics were also proposed, including diabetes in general, symptoms of hypoglycemia versus epilepsy, and hyperglycemia.

**Table 7.** Example Comments From Two Three-Hour Focus Group Sessions and a Web Survey, Indicating Preferences Regarding the Different Presentation Modes of the Three Prototypes.

Likes/dislikes	Example feedback
Prototype 1	
• *Most preferred* prototype: favorite avatar, favorite environment, explains the concept best • Enjoyed the 3D immersive VR element • Visuals were relatable and familiar • Inside the bloodstream is incredibly visual, engaging, and aids understanding • The content looks too distant • Some participants had issues viewing this content	• “The guided tour of the bloodstream in 3D is what I liked the most, super interesting. I did not imagine it this way” • “The 3D prototype is more immersive, you see the red blood cells, the adverse effects, the explanation, the scale is very explanatory” • “The use of virtual reality making the experience more immersive” • “Because it is three-dimensional, very striking, which makes it focus on the information, and not be monotonous” • “I like that environment because it seemed more pleasant, familiar.” • “Prototype 1 is the most complete, you stay immersed and it makes you concentrate” • “The headset is important for concentration. The novelty of getting into the bloodstream and seeing the red blood cells, it’s compact and well done” • “It looks real, like a human being, like you or me”; “More android”
Prototype 2	
• *Second preference*, tying with prototype 3 • Respondents enjoyed the animation and thought that the video was eye-catching • The void space is less distracting, drawing more attention to the visuals • The robot is very impersonal	• “More eye-catching for a 3d video” • “I liked the robot as it enters to see the red blood cells, it is very striking” • “I don’t want to think that I’m a robot” • “Very impersonal. Far from your reality” • “He did not identify me with that character” • “Prototype 2 is best when you don’t have the Google Cardboard headset”
Prototype 3	
• *Second preference*, tying with prototype 2 • People identified with the characters and found the content easy to understand • The content format is less original	• “I identified more with this option” • “It is easier to understand” • “It is something very common” • “Prototype 3 is more educational with a very striking background”

## Discussion

The main goal of this QI study was to evaluate the potential of an avatar-based program to educate PWD in Colombia. Hypoglycemia reduces health-related quality of life^26,27^ and 25% of PWD struggle with hypo-anxiety.^21^ Importantly, many hypoglycemic events are avoidable, so an educational intervention could be highly effective. The methodology relied on collaborative working with Colombian clinicians and PWD, and the use of a structured method, all of which are essential components for effective QI.^28^ The results suggest that, overall, immersive video technology has a high level of acceptability as an educational intervention, both by PWD and by expert clinicians.

The feedback on all three prototypes was enormously positive. Survey data supported our hypothesis that immersive video technology could be an appealing format, and that participant “buy-in” was not necessarily an obstacle for this pilot to overcome. All versions were considered to be very effective educational tools, with potential to educate even beyond the clinical setting. The 3D video with a realistic avatar was the most highly rated, and the need for such engaging, remote education was reinforced by the pandemic.

People were responsive to the prospect of educational intervention and believed that positive lifestyle changes can have a beneficial impact on outcomes. The curriculum topics were useful, but future work should consider covering vision, self-care, and emotional support. Some additional issues emerged, such as time limitations of PWD. The technical delivery went very smoothly: most people could access the content easily on their phones. The results indicate that even these short three-minute prototypes may have positive influence on quality of life and confirm the need to empower PWD through knowledge, perhaps as a priority for T2D, where care may be more oriented toward reducing hyperglycemia.^29^

The study was not without its challenges. The COVID-19 pandemic meant that all activities had to be moved online, incurring a delay in the approval of the amended ethics submission. The pandemic also intensified the HUSI clinicians’ workload. The online execution did have the advantage that Spanish-speaking investigators based in the United Kingdom could participate, however. There were also logistical challenges: COVID-19 disrupted the Colombian postal services, leading to delays in delivery of the Google Cardboard headsets. Language introduced another obstacle as all research materials needed to be translated to Spanish and vice versa for the data analysis. Fortunately, the multidisciplinary nature of the team made it highly adaptable to overcoming such development challenges.

This research does have limitations as it is based on feedback from one hospital (HUSI) in one region of Colombia (Bogotá). Results were self-reported, the study size was small, and the cohort included many people with a high level of education and long diabetes duration. Nevertheless, this QI study demonstrates how immersive technology has potential for use either in clinical settings or in diabetes education centers, as an adjunct to current educational practices. The educational prescription model in Figure 2 outlines the simple, practical approach that could be used, for example, to provide basic information, thereby facilitating health care practitioners to focus on different content.

## Conclusions

The results of this work show that it is possible to conclude that immersive video technology has the potential to deliver patient education in Colombia, through smartphone. The human-centered design methodology proved vital in understanding the target audience and their perceived needs. The work also highlighted the importance of involving experienced diabetes-specialist clinicians in the content development process to ascertain their expert perspective of individual health education needs.

Although existing research has demonstrated the benefits of using VR to educate clinical practitioners,^15,30^ this is the first report of a 3D avatar-based diabetes education program for patients in Colombia. One reason for this could be the prohibitive cost of deploying such an intervention at scale, or unfamiliarity with the appropriate equipment. These barriers have been removed by delivering the program through smartphone, together with an affordable headset. The results are timely as the pandemic has exacerbated the need for such digital, remote, and instructive technology. Future work includes development of additional content, evaluation with a larger group using validated tools, and exploration of the role of artificial intelligence in personalizing this form of education.
